# Patient-perceived acceptability of a virtual world-based cardiac rehabilitation program

**DOI:** 10.1177/2055207617705548

**Published:** 2017-04-24

**Authors:** LaPrincess C Brewer, Brian Kaihoi, Karen Schaepe, Kathleen Zarling, Ray W Squires, Randal J Thomas, Stephen Kopecky

**Affiliations:** 1 Department of Cardiovascular Medicine, Mayo Clinic College of Medicine, USA; 2 Global Products and Services, Center for Innovation; Mayo Clinic, USA; 3 Qualitative Research Services; Mayo Clinic Center for the Science of Health Care Delivery, USA; 4 Department of Nursing, Mayo Clinic, USA

**Keywords:** Cardiac rehabilitation, cardiovascular diseases, eHealth, telemedicine, Internet, health behavior, virtual systems, virtual environments, home-based programs

## Abstract

**Background:**

Despite its benefits, cardiac rehabilitation (CR) participation rates remain subpar. Telehealth lifestyle interventions have emerged as modalities to enhance CR accessibility. Virtual-world (VW) technology may provide a means to increase CR use.

**Objectives:**

This pilot study assessed the feasibility and acceptability of a VW-based CR program as an extension to medical center-based CR. Our goal is to apply the study results toward the design of a patient-centered VW platform prototype with high usability, understandability, and credibility.

**Methods:**

Patients (*n* = 8, 25% women) recently enrolled in outpatient CR at Mayo Clinic, Rochester, Minnesota participated in a 12-week, VW health education program and provided feedback on the usability, design and satisfaction of the intervention at baseline and completion. A mixed-methods approach was used to analyze the participant perceptions of the intervention.

**Results:**

Overall, there were positive participant perceptions of the VW experience. There was unanimous high satisfaction with the graphical interface appearance and ease of use. Participants placed value on the convenience, accessibility, and social connectivity of the remote program as well as the novelty of the simulation platform presentations, which aided in memorability of key concepts. Greater than 80% of participants reported that the program improved their health knowledge and helped to maintain better health habits.

**Conclusions:**

Our pilot study revealed the feasibility and acceptability of an innovative VW-based CR program among cardiac patients. This novel delivery method for CR has the potential to influence healthy lifestyle change and to increase accessibility to vulnerable populations with higher cardiovascular disease burdens.

## Introduction

Despite an abundance of evidence demonstrating clear benefits of cardiac rehabilitation (CR) for patients with coronary artery disease and other cardiac diagnoses in improving cardiovascular fitness, cardiovascular disease (CVD) risk factors and quality of life, and reducing hospitalizations and cardiovascular mortality, it remains drastically underused.^1,2^ The reasons for this issue are multifactorial, inclusive of patient-, provider-, and systems-level factors.^3^ The current CR model must undergo significant infrastructure changes focusing on increasing patient convenience through alternative delivery models. Approaches to “rebrand and reinvigorate” traditional CR programs through the integration of digital and mobile technologies are key to transforming the current paradigm toward a more patient-centered program to increase CR enrollment, participation, and completion.^2,4–8^

Fundamental strategies to integrate into CR programs include those that promote social connectedness, engagement, and quality of life.^9^ Virtual-world (VW) environments offer a potential innovative technology to improve health outcomes through rehabilitation,^10,11^ disease self-management,^11–13^ healthy lifestyle change^14^ and social networking.^9^ VWs are immersive, three-dimensional environments that foster a more synchronous experience for experiential learning, skill-building, and socialization.^10^ These inherent features are patient-centric and complementary to CR core components and competencies as outlined by national secondary prevention guidelines that aim to optimize cardiovascular risk reduction, foster healthy behaviors, and promote an active lifestyle for cardiac patients.^15^ VWs may also represent a valuable tool to expand the reach of CR by increasing its accessibility to underserved groups.^10,12,13^

The aims of this pilot study were the following: (1) to assess the feasibility of implementation of a 12-week VW-based CR program as an extension to conventional CR, (2) to probe the acceptability, ease of use, utility, and satisfaction of a VW-based CR education program among cardiac patients, and (3) to examine the perceptions regarding the VW-based platform by cardiac patients. The information gleaned from the study will inform the design of a patient-centric, VW platform prototype with high usability, understandability, and credibility to apply within a broader comparative effectiveness study.^10^

## Methods

### Study setting and participants

We recruited patients recently enrolled in outpatient CR at Mayo Clinic Rochester, Minnesota, through the assistance of CR staff. Among those recruited, the indications for CR were the following: recent hospitalization for acute coronary syndrome (ACS) (unstable angina, ST-segment elevation myocardial infarction, non-ST-segment elevation myocardial infarction), heart valve replacement, elective percutaneous coronary intervention (PCI), and stable angina. Eligible patients were required to continue with standard CR and have regular high-speed internet access (home, work or community). Patient exclusion criteria included < 18 years of age, lack of basic internet navigation skills and non-fluency in English. The feasibility study research protocol was reviewed and approved by the Mayo Clinic Institutional Review Board.

Upon informed consent completion, each participant received hands-on instructional training by a study investigator (B.K.) on the VW platform (Second Life® account activation, avatar creation, basic navigational skills). Personal laptops installed with VW software and personal headsets were provided to each participant for use throughout the intervention.

### Intervention

#### Intervention development

The study investigators engaged with two community-based, cardiac patient-led groups in intervention development. One group, One Voice, is a patient/family advisory council within the Mayo Clinic Department of Cardiovascular Diseases, which provides input on any aspect of practice, education, research, policy, and procedure development to ensure responsiveness to individual patient values and preferences. The other group, the Rochester Coronary Club, is a local support group for CVD survivors. Three community presentations detailing the feasibility study proposal were delivered by the study team (L.B., B. K., S.K.) with integrated VW (Second Life®) platform demonstrations from January 2014 to February 2015. The proposal was well received by both groups and the study team gleaned excellent feedback on recruitment strategies and intervention refinement.

Self-determination theory^16^ informed the development of the VW platform and education curriculum to foster healthy behavior change given its focus on an individual’s beliefs and effectiveness in performing behaviors (competence), connection with others (relatedness), and perceived control over behaviors (autonomy). In addition, the intrinsic features of the intervention are to encourage healthy lifestyle behaviors by participant avatars with the goal of transferring these behaviors to the real world (i.e. Proteus effect^14^).

#### Intervention delivery

**Figure 1.**
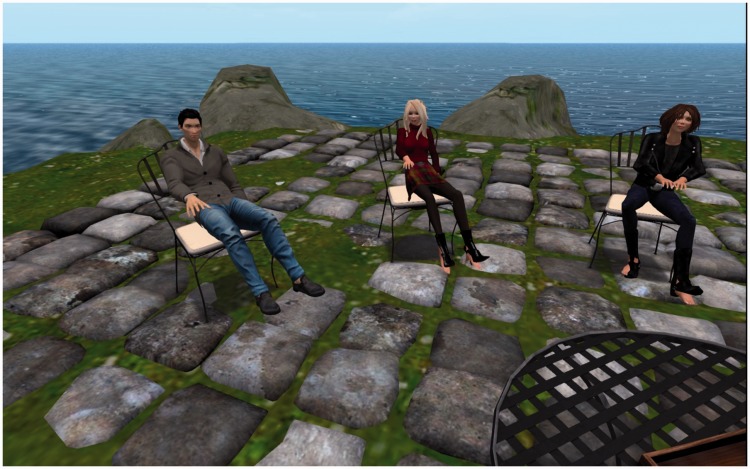
Peer social support group, concluding education session 12.

Participants engaged in a 12-week education session series delivered by expert health professionals specializing in CR delivery on a secure VW platform via an established Mayo Clinic infrastructure on Linden Lab’s Second Life®. These included comprehensive topics on cardiovascular health behaviors (physical activity, diet), cardiac risk factors (smoking, hypertension, hyperlipidemia, etc.), and those most relevant to CR (sexuality and heart disease, heart medications) as endorsed by the American Heart Association (AHA), American College of Cardiology (ACC), and Association of Cardiovascular and Pulmonary Rehabilitation (AACVPR) (see Table 1).^15^ Interactive, group virtual “field trips” were integrated into the program including restaurant and fitness center tours with a dietician and exercise physiologist. The concluding session was a support group for reflection on the VW experience and personal journey with CVD (see Figure 1). There were 12 sessions total delivered in a variety of formats including lectures, group tours, and discussion forums. Live sessions were held once weekly for 1.5 hours on a consistent weekday afternoon identified as most convenient by the study participants at enrollment. The sessions were led and moderated by a CVD specialist (L.B.). Technical support staff (B.K.) was present at each session and provided troubleshooting assistance to speakers and participants. Each session was structured with an introduction facilitated by the CVD specialist that included reflections from participants on their VW experience, lessons learned from the prior session, and integration of healthy lifestyle practices. This was followed by an introduction to the current and subsequent session topics and speakers that then advanced to the education session. To maintain confidentiality of our study participants, no protected health information was collected on the VW platform itself. Furthermore, for additional security, the VW platform was password protected and open only to the study participants and study team.

**Table 1.** Virtual world education session topics.

Session	Topic
1	Managing heart disease risk factors
2	Heart medications
3	Benefits of exercise
4	Smoking cessation
5	Practical exercise tips
6	Stress management and heart disease
7	Sexuality and heart disease
8	Heart healthy nutrition review
9	High blood pressure
10	Fitness concepts and implementation strategies (including fitness center tour with exercise physiologist)
11	Dining out the healthy way (including interactive restaurant tour with dietician)
12	Peer social support group

### Measures

A comprehensive electronic pre-intervention survey assessed participant sociodemographics, social support,^17^ digital health information access, and prior VW experience. The post-intervention survey included 18 questions probing participant perceptions of the VW intervention on several domains (overall experience, usability, logic of information, and utility) through both closed and open-ended questions from previously validated tools.^18–22^ Participants rated each domain on a five-point Likert scale (very unsatisfied to very satisfied or strongly disagree to strongly agree). We aggregated responses into “satisfied/very satisfied,” “neutral,” “strongly disagree/disagree,” and “agree/strongly agree” as a percentage of the total participant group. Participant education session series attendance and presentation feedback (style, content, etc.) statistics were also collected.

### Data analysis

#### Quantitative data

For normally distributed variables, simple arithmetic means and standard deviations were calculated. Frequencies and proportions were calculated for categorical variables. Analyses were performed using commercial software (SAS, version 9.2; SAS Institute).

#### Qualitative data

Qualitative analysis was conducted by an independent analyst (K.S.) of the participant post-intervention open-ended responses that was informed by both ethnomethodology^23^ and structural narrative analysis^24^ approaches. Ethnomethodology is a descriptive approach to understanding behaviors among individuals within groups and how they use social interaction to maintain an ongoing sense of reality in situations and environments. This framework facilitated the systematic review of the narrative responses according to their structure and conveyed meanings. Initially, a comprehensive catalog of aggregated major themes of likes, dislikes, and suggestions for improvements to the VW platform was created. Subsequently, a form of structural narrative analysis was used to examine how each respondent connected themes into a sequence to convey meaning about what mattered most or least or in relation to another feature of the VW platform.

## Results

### Sample description

Participant baseline characteristics are presented in Table 2. Of the eight participants enrolled, seven participated in the education session series. Participants were predominately white (87.5%) males (75%) with a mean age of 67. The majority were college-educated, retired, and married. Most participants had also undergone a PCI (6/8) either emergently with an ACS or electively. Eighty-eight percent reported using university/hospital websites to access health information and 63% were unaware of VW technology prior to the study.

**Table 2.** Baseline characteristics of study participants (*N* = 8)^a^.

Characteristic	
Age, mean (SD), years	67.3 (7.1)
Women, n (%)	2 (25%)
Education level, *n* (%)	
College graduate	5 (63%)
Marital status, *n* (%)	
Single	1 (13%)
Married	5 (63%)
Separated/divorced/widowed	2 (25%)
Employment status, *n* (%)	
Retired	7 (88%)
Full time/part time	1 (13%)
Annual household income, *n* (%)	
<US $50,000	2 (25%)
US $50,000–$100,000	4 (50%)
>US $100,000	2 (25%)
Health insurance status, *n* (%)	
Insured	8 (100%)
Self-reported medical history, *n* (%)	
Diabetes	1 (13%)
Hypertension	5 (63%)
Hyperlipidemia	4 (50%)
Current tobacco use	0 (0%)
Cardiac rehabilitation indication, *n* (%)	
Acute coronary syndrome	3 (37.5%)
Percutaneous coronary intervention	6 (75%)
Heart valve replacement	1 (12.5%)
Stable angina	1 (12.5%)
Self-reported health status, *n* (%)	
Poor–Fair	3 (38%)
Good–Excellent	5 (63%)
Social support^b^, mean (SD)	29.0 (5.4)
Health information source, *n* (%)	
Hospital/University websites	7 (88%)
Awareness of virtual world or Second Life® prior to study, *n* (%)	
No	5(63%)

a A total of eight participants enrolled into the study; one participant withdrew before the start of education session series.

b Social support is represented as an average of a total score sum of seven items from each participant.^11^ (Items 1–6, Scale 1–5: 1 [“None of the time”] through 5 [“All of the time”] and Item 7 scored 4 for “yes” and 2 for “no.”; score range: 8–34).

### Intervention acceptability

Participants reported overall positive perceptions of the VW-based CR education program are presented in Tables 3 and 4. All participants (100%, 6/6) were highly satisfied with the global VW experience in terms of ease of locating, reading, and listening to information housed on the VW platform. There were also positive impressions of the graphical interface appearance. Perceived usefulness was highly rated (agree/strongly agree) in terms of understanding specific health problem information (67%, 4/6), gaining health knowledge (83%, 5/6), and maintenance of better health habits (67%, 4/6). The process of information-gathering was also logical and simple to more than half of the participants. As a composite of all 12 education sessions, participants ranked the style, content, and presentation aids as very good to excellent with a concordant overall scoring. Each participant attended on average eight of the 12 sessions (67% of the entire education session series) with an average attendance of five participants at each session (71% of those enrolled).

**Table 3.** Virtual world-based cardiac rehabilitation acceptability (*N* = 7).

Domain	*n* (%)	
Overall experience		
Ease of finding specific information		
Satisfied/Very satisfied	6	(100%)
Ease of reading the information given		
Satisfied/Very satisfied	6	(100%)
Ease of listening to audio information		
Satisfied/Very satisfied	6	(100%)
Overall appearance of the platform		
Satisfied/Very satisfied	6	(100%)
Overall quality of the graphics		
Satisfied/Very satisfied	6	(100%)
Usability		
I found the use of this platform easy to learn		
Neutral	1	(17%)
Agree/Strongly agree	5	(83%)
Finding information on this platform requires a lot of mental effort		
Strongly disagree/disagree	4	(67%)
Agree/Strongly agree	2	(33%)
Overall, I find this platform easy to use		
Agree/Strongly agree	6	(100%)
Logic of information		
In this platform, finding information is a logical and simple process		
Neutral	2	(33%)
Agree/Strongly agree	4	(67%)
All applications in this platform can be carried out in a similar manner		
Strongly disagree/disagree	2	(33%)
Agree/Strongly agree	4	(67%)
Utility		
Understand specific health problems		
Neutral	2	(33%)
Agree/Strongly agree	4	(67%)
Improve my knowledge about health		
Neutral	1	(17%)
Agree/Strongly agree	5	(83%)
Maintain better health habits		
Neutral	1	(17%)
Agree/Strongly agree	5	(83%)

a Frequencies not adding up to post-intervention total (*N* = 7) indicate missing data.

**Table 4.** Virtual world-based education session series feedback.

Variable^a^	*n* (%)^b^	
Style		
Poor/Fair	1	(1%)
Good	16	(18%)
Very good/Excellent	52	(57%)
Content		
Poor/Fair	2	(2%)
Good	15	(16%)
Very good/Excellent	52	(57%)
Presentation aids		
Good	15	(17%)
Very Good/Excellent	53	(59%)
Overall score		
Poor/Fair	1	(1%)
Good	15	(16%)
Very good/Excellent	53	(58%)

a For each variable, percentages not adding up to 100% represent missing data or participant lack of attendance of particular sessions.

b Note frequencies represented as composite of all participants (*N* = 91 responses).

### Intervention qualitative perceptions

Post-intervention survey responses revealed several themes regarding likes, dislikes, and suggestions for improvement of the intervention and education program. These responses coalesced into three valued priorities among the participants including: (1) time/space flexibility, (2) memorability, and (3) post-intervention concept reinforcement.

#### Time/space flexibility

Participants specifically liked that the program could be accessed anywhere, even while traveling, and did not require preparation. One individual noted:

“It is interactive with visuals yet can be done from home and without having to take the time to comb my hair (or other embarrassments of face time) …it’s easier to pick up a computer for an hour at home than to get dressed, get in the car and go somewhere for an hour session somewhere away from home which ends up taking two hours when it is all done.”

Another point of emphasis with the VW experience was the ability it afforded to interact and focus during the education session presentations without the typical distractions of face-to-face CR sessions. For example, one participant expressed:

“One of the best parts of Second Life was the ability to interact, ask questions, to participate. Without the distraction of evaluation that comes with seeing another person. Or the self-consciousness of being seen.”

However, one disadvantage noted of the VW education session series was the inability of presenters to adjust their presentation styles based on audience gestural feedback (i.e. slow presentation speed):

“Some speakers started to rush through as the talk went along as if they thought the audience had quit listening. They might have had better presentations with eye contact.”

#### Memorability

Participants felt that the VW simulations and range of physical environments (restaurant, exercise facilities) intensified the emotional experience and, thus, enhanced the memorability and retention of the subject matter on healthy lifestyles. One participant mentioned that he or she had developed an emotional connection to their avatar, noting that the VW technology created a sense of really “being there,” of “humanity,” and being “person-to-person”:

“I would miss that in a way that would not be true in any other online interaction.”

Another commented that hearing testimonials of others deepened their sense of emotional connection to people physically remote, arguably making the VW material more relevant and impressive:

“Everyone getting to know each other and speaking up … to communicate with the presenter, staff and fellow participants and hearing other participants’ stories and opinions.”

Participants particularly found the social support group to be memorable and suggested scheduling this as the first session of the education session series.

#### Post-intervention concept reinforcement

Participants expressed a desire to connect the series to real life; that is, to reinforce learning from the education sessions to life beyond the program. Several expressed a strong desire to build community beyond the workshop, with use of the VW platform for forums to mentor and support each other. An illustrative comment promoting social support is as follows:

“Invite me back! Teach me how to be a support layperson counselor so that I can help others be more comfortable with this virtual world experience.”

Although the VW platform features endorsed new ways to adopt healthy behaviors, participants acknowledged the importance of translating these practices to the real world and recommended emphasis on this crucial concept in future programming. Participants also suggested supplemental resources including an “online bulletin board” to allow for continued discussions on specific education sessions, the receipt of “abstracts” prior to presentations for preparation, and the ability to download the presentation slides after the education sessions for reinforcement of key concepts. Furthermore, self-directed learning was encouraged outside of the scheduled education sessions through an “ongoing program” with a monthly rotation of topics.

Beyond the aggregated three priorities, additional comments highlighted the novelty of the VW delivery method for CR:

“I cannot think of any other application or situation that offers all these things, making it unique … I think there are endless possibilities for this format.”

Several participants offered further ideas for expansion of the VW curriculum and platform to foster ongoing learning opportunities, which speaks to the enthusiasm participants had following the program. These were inclusive of a three-dimensional human heart tour (with displays of various heart diseases), simulations of cardiac catheterization procedures, demonstrations of a variety of exercises (i.e. physical therapy, yoga, tai-chi), healthy cooking demonstrations, and self-monitoring mechanisms. One participant wanted the program to continue, stating a main dislike that it was “over,” further underscoring the program’s overall positive perception and acceptability.

## Discussion

Capitalizing on telemedicine advances toward creating alternative CR delivery models is paramount to increasing CR access and participation.^2^ Our study results suggest the potential use of VW technology as a feasible and acceptable means for promoting CR participation and delivering cardiovascular health education. Participants reported overall positive perceptions of the VW-based CR program due to its ease of use, innovative graphics and simulations, and novel education delivery approaches. Many participants felt that the interactive simulations and real-time VW education sessions aided in their memorability of key concepts of secondary CVD prevention, which prompted adoption of healthy behaviors in the real world—the ultimate objective to improve health outcomes (i.e. Proteus effect).^14^ Participants reported a minimization of distractions within the VW environment in comparison to the face-to-face environment of center-based CR that allowed them to focus on the CR education content and develop supportive, virtual interactions with other participants. Participants particularly gravitated to the social connectivity and sense of camaraderie afforded by the VW experience in coping with shared experiences of heart disease.

We recognize that our pilot study is a small, single-site feasibility trial that limits generalizability. We also acknowledge that our participants were largely of high socioeconomic status; however, the majority lacked prior experience with VW technology. VW technology has also been successfully used within a socioeconomically disadvantaged group (of variable digital literacy) engaged in a diabetes self-management program with high user satisfaction and retention.^13^ Nonetheless, it is the first study to our knowledge evaluating the use of VW-based technology for CR, particularly through a designated medical center of excellence. Our study indicates an openness and perceived utility toward a VW environment for home-based CR among cardiac patients that has been further supported by similar groups.^25^ Participant engagement and retention were excellent as the education sessions were well-attended and all but one participant completed the entire program. Future studies using VW in CR should emphasize social support and networking to promote participant cohesiveness and relationship-building. It is also of utmost importance to assess the acceptability of VW in CR among underserved groups with the lowest CR utilization, including ethnic minorities, rural residents, the elderly, and the economically disadvantaged. Thus, recruitment is underway for the next phase of the VW in CR trial by the study investigators with an aim of prioritizing this patient demographic.
